# Self-diagnosed depression in the Norwegian general population – associations with neuroticism, extraversion, optimism, and general self-efficacy

**DOI:** 10.1186/s12889-018-5990-8

**Published:** 2018-08-29

**Authors:** Tore Bonsaksen, Tine K. Grimholt, Laila Skogstad, Anners Lerdal, Øivind Ekeberg, Trond Heir, Inger Schou-Bredal

**Affiliations:** 1 Department of Occupational Therapy, Prosthetics and Orthotics, Faculty of Health Sciences, OsloMet - Oslo Metropolitan University, PO Box 4, St. Olavs Plass, 0130 Oslo, Norway; 2 Faculty of Health Studies, VID Specialized University, Sandnes, Norway; 3 Department of Acute Medicine, Oslo University Hospital, Oslo, Norway; 4 Department of Nursing and Health Promotion, Faculty of Health Sciences, OsloMet - Oslo Metropolitan University, Oslo, Norway; 5 Department for Patient Safety and Research, Lovisenberg Diakonale Hospital, Oslo, Norway; 6 Department of Nursing Science, Institute of Health and Society, Faculty of Medicine, University of Oslo, Oslo, Norway; 7 Division of Mental Health and Addiction, Oslo University Hospital, Oslo, Norway; 8 Department of Behavioural Sciences in Medicine, University of Oslo, Oslo, Norway; 9 Norwegian Center for Violence and Traumatic Stress Studies, Oslo, Norway; 10 Institute of Clinical Medicine, University of Oslo, Oslo, Norway; 11 Institute of Health and Society, University of Oslo, Oslo, Norway; 12 Department for Cancer, Oslo University Hospital, Oslo, Norway

**Keywords:** Extraversion, Gender, General self-efficacy, Life orientation test, Neuroticism, Optimism, Personality, Population study, Self-report, Survey

## Abstract

**Background:**

Multi-item rating scales for depression informs about the level of depression, but does not allow individuals to state by self-evaluation whether they feel depressed or not. The insider perspective on depression is rarely assessed. This study investigated the prevalence of self-diagnosed depression in the Norwegian general population, and associations with sociodemographic and psychological factors.

**Methods:**

As part of a national survey, the General Self-Efficacy Scale, the Life Orientation Test-Revised, a short version of the Eysenck Personality Questionnaire and a one-item measure of self-diagnosed depression was administered to 5.500 persons in the general Norwegian population. Of the 4961 eligible participants ≥ 18 years of age, 1.787 (response rate 36%) participated in the survey, and 1.684 of these had valid scores on the relevant scales. The associations between sociodemographic factors and self-diagnosed depression were examined using univariate and multivariate logistic regression analyses.

**Results:**

One hundred and thirty-six participants (8.1%) reported depression during the preceding month. When adjusting for sociodemographic and psychological variables, higher age (OR = 0.82), being in work (OR = 0.57), and higher levels of general self-efficacy (OR = 0.67) and optimism (OR = 0.52) were associated with lower risk of self-diagnosed depression, whereas higher levels of neuroticism (OR = 1.97) was associated with higher risk.

**Conclusions:**

The prevalence of self-diagnosed depression in the adult Norwegian population was higher for women than for men. Higher age, being in work and having higher levels of psychological resources appear to reduce the risk of self-diagnosed depression, whereas neuroticism increases the risk.

## Background

Norway is the world’s happiest country, according to the 2017 World Happiness Report, and the country is ranked highest in standard of living**,** life expectancy, and education [1]. However, the high life expectancy is not necessarily linked to better health [2]. Depressive disorders, as defined by the DSM-5 [3], is rated among the top 10 disorders in Norway causing disability [2]. According to the World Health Organization (WHO), depression is one of the most burdensome disorders worldwide, robbing people of more healthy years than any other illness in the western world [4]. Depression is common and frequently chronic, and it is associated with substantial costs for individuals as well as for society. Worldwide, depression has an estimated average prevalence of 4.4%, with proportions being higher for women (5.1%) than for men (3.6%) [5]. Societal factors appear to influence depression rates, as the lifetime prevalence of depression is reported to be higher in high income countries (14.6%) compared to low- and middle income countries (11.1%) [6].

Personality traits may also, directly or indirectly, influence both somatic and mental health outcomes [7]. Personality traits such as high neuroticism and low extraversion have been found to be strongly associated with depression in both general and clinical populations [8, 9]. Neuroticism is characterized by proneness to anxiety, emotional instability and self-consciousness, whereas extraversion involves positive emotionality, energy, and dominance [10]. Similarly, dispositional optimism has been found to be negatively associated with depression [11, 12]. Dispositional optimism describes the degree to which a person generally expects positive outcomes [7]. Further, lower self-efficacy has been associated with higher depression levels [13]. Self-efficacy denotes a person’s confidence that they are able to perform the behaviors needed to bring about desired outcomes. Thus, self-efficacy contributes to determine how people feel, think, and behave [14].

In addition to personality traits, studies have reported gender to be associated with depression. Higher prevalence of depression among women compared to men has been a relatively consistent observation among adults in the general population [15–17], although the opposite has also been found [18]. Some studies have found significant associations between depression, neuroticism and female gender [19, 20], and a recent study found associations between lower age, male gender and higher general self-efficacy in the Norwegian general population [21]. In contrast, no gender or age difference was found in dispositional optimism in the general population [22]. Few studies have investigated depression and associated personality factors in general populations. In one such study from Finland, higher levels of neuroticism and lower levels of extraversion were found to be associated with higher levels of depression, when controlling for sociodemographic characteristics [9].

Studies of depression in various samples seem to have largely focused on associations with personality traits in isolation, rather than examining several personality traits in combination (e.g., [9, 23, 24]). Such traits might have potential to moderate each other’s association with depression [25]. Moreover, in addition to structured diagnostic interviews, different methods have been used for assessing depression. For example, multi-item depression rating scales provide information about the level of depression. On the other hand, self-evaluation methods allow individuals to state whether they feel depressed or not. Despite having received criticism related to measurement properties, one-item self-evaluation measures have the advantage of being short, flexible, and easy to use [26], compared to multi-item scales [27]. The degree of concurrence between a clinical diagnosis of depression and self-diagnosed depression appears to be mixed. Some have found a high degree of concurrence (85%) between the two [28], whereas others have found self-diagnosed depression to be more frequent than clinically diagnosed depression [29]. As a result, one might expect somewhat higher prevalence rates of depression when the results are based on self-evaluation rather than diagnostic assessment. In this study, we assessed self-diagnosed depression, keeping with an insider perspective in our investigation of depression and its related factors.

### Study aims

The aims of the present study were to investigate (i) The prevalence of self-diagnosed depression in the general Norwegian population, and (ii) Associations between sociodemographic variables, personality and self-diagnosed depression.

## Methods

### Study design

The Norwegian Population Study (NorPop) is a cross-sectional survey design study. The collected data reflects a wide variety of health conditions in the general population, and the data will provide national norm scores related to several questionnaires used for assessing symptoms, attitudes and behavior. For more detailed description of the survey methodology, see Schou-Bredal and colleagues [22].

### Sample selection

A random sample of 5500 adult persons in Norway (inclusion criterion ≥18 years of age), proportionately stratified by age, gender and geographic region (including both urban and rural regions from the whole country), was selected for inclusion in the study. The randomized selection was performed by an external agency, the National Population Register. The questionnaires were sent by regular mail to all the invited individuals along with a letter explaining the purpose and procedures of the study.

**Fig. 1:**
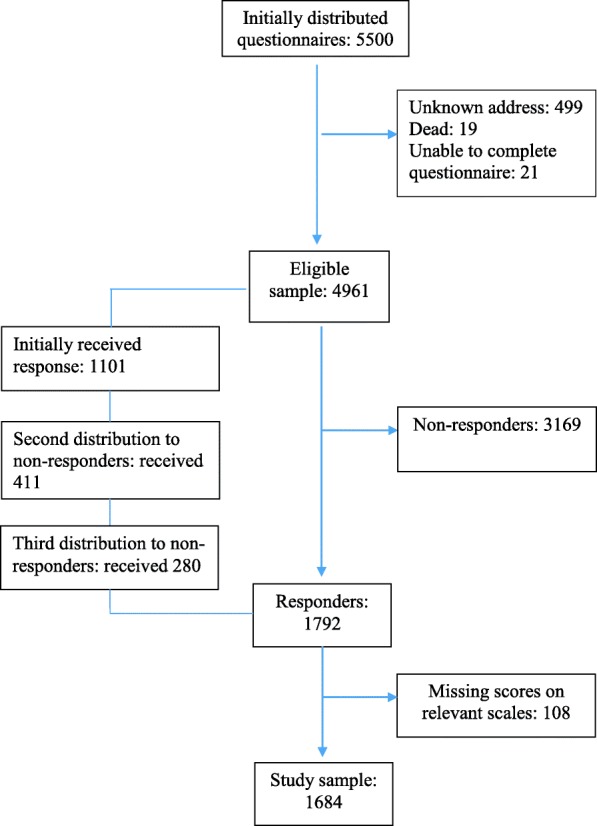
Flowchart showing the inclusion of the participants

There were no significant differences in mean age, gender proportions or the distributions of living in rural and urban areas between responders and non-responders. The proportion of study participants working was 66%, compared to 67% in the general population [30]. Seventeen percent lived alone, in both groups. Among the study participants, 1.3% were without work and 53% had higher education, compared to 4.4% and 41.0% in the general population [22]. Thus, in terms of relationships status, work status and education level we consider our sample fairly representative of the general Norwegian population, although in the sample a somewhat larger proportion had higher education. The flowchart in Fig. 1 displays the recruitment and inclusion process. All data were collected in 2015 and 2016.

### Measures

Research has demonstrated that single-item measures are viable alternatives to multi-item scales for measuring constructs [31]. Mahoney and colleagues [28] showed that affirming responses to a single screening question for depression (“Do you often feel sad or depressed?”) corresponded with a clinical diagnosis of depression in 85% of the cases. Single-item measures of self-rated mental health are increasingly used in health research and population health surveys, as they reduce the burden for the respondents compared to longer scales [32, 33]. Thus, a single-item measure of depression was used in the present study. In order to conduct a sensibility test for self-diagnosed depression, we also investigated the proportion of those with reported depression who had also sought help for their mental health problems.

#### Self-diagnosed depression and help seeking

For the purpose of the present study, we used the following phrase: “Below you will find listed some mental health problems. Do you have, or have you had, any of these problems?” Depression was one of the listed problems. The response alternatives were “no”, “yes previously, but not during the last month” and “yes, during the last month”. Current self-diagnosed depression (reported as depression during the last month) was coded 1 (current depression), whereas no previous depression or depression prior to the last month was coded 0 (no current depression). Lifetime self-diagnosed depression was registered for those who reported depression as a previous or current mental health problem. Similar one-item self-diagnostic measures of depression have been shown to correspond well with a clinical diagnosis of depression, indicating good construct validity of the one-item measure [34, 35]. In the current sample, no clinical diagnoses were available. However, the correlation between self-diagnosed depression and general perceived health during the last week (assessed with an 11-point rating scale, 0 = very poor, and 10 = excellent) was *r* = − 0.31 (*p* <  0.001). Similarly, the correlation between self-diagnosed depression and optimism (assessed with a 24-point rating scale, 0 = very pessimistic, and 24 = very optimistic) was *r* = − 0.23 (*p* <  0.001), both results indicating discriminant validity of the depression measure.

The respondents were further asked: “Have you sought help for your mental health problems”, with the response alternatives “no, not applicable”, “no, but I plan to do so”, or “yes”. Respondents indicating “yes” were then prompted to indicate from whom (general practitioner, psychologist, psychiatrist, district psychiatric center) they had sought help for their mental health problems, currently or previously.

#### General self-efficacy

The General Self-Efficacy Scale (GSE) [36] measures self-beliefs related to coping with the demands, tasks, and challenges of life in general. Respondents rate the 10 GSE statements from 1 (not at all true) to 4 (exactly true). Examples of statements are “I can always manage to solve difficult problems if I try hard enough” and “I am certain that I can accomplish my goals”. For the present study, the GSE score was calculated as the mean of all item scores, ranging from 1 to 4, where higher scores indicate higher general self-efficacy. Factor analyses of the GSE have consistently produced a one-factor solution, which was confirmed in a previous study with the Norwegian general population [21]. Cronbach’s α was 0.92.

#### Optimism

The *Life Orientation Test - Revised* (LOT-R) was used to measure dispositional optimism [37]. The LOT-R consists of 10 self-reported items, where four items are distractors used to disguise the purpose of the measure. Of the remaining six items, three are phrased in an optimistic and three in a pessimistic direction. An example of an optimistic statement is “In uncertain times I usually expect the best”, whereas a pessimistic statement example is “If something can go wrong for me, it will”. The respondents indicated the extent to which they agreed with each of the items on a 5-point scale from 0 (*strongly disagree*) to 4 (*strongly agree*). For the present study, the total LOT-R score was calculated as the mean of the optimism and pessimism item scores, with the pessimism scores inverted. Thus, scores ranged from 0 to 4, with higher scores indicating more optimism. Factor analysis supported that the LOT-R can be used with a one-factor structure, and Cronbach’s α for the one-factor measure was 0.75 [22].

#### Personality

The *Eysenck Personality Questionnaire* (EPQ) is a self-report questionnaire designed to assess personality traits [38]. In line with the third ‘Helseundersøkelsen i Nord-Trøndelag’ (the HUNT-3 study) [39], we used a shortened version of the EPQ, omitting the psychoticism scale. Thus, the EPQ assessed two dimensions of personality: extraversion (degree of liveliness and social orientation) and neuroticism (dispositional worry and nervousness), each assessed with six questions to which the respondent was asked to circle “yes” or “no”. Example statements are “Do you like to meet new people?” (extraversion), and “Are your feelings easily hurt?” (neuroticism). Higher sum scores on each of the scales, both ranging from zero to 6, would indicate higher levels of extraversion and neuroticism, respectively. Factor analysis differentiated clearly between the two underlying dimensions, supporting the validity of the scales. Cronbach’s α was 0.76 for the extraversion scale, and 0.77 for the neuroticism scale.

#### Sociodemographic background

Data regarding age, sex, education, and employment status were collected. The age variable was transformed into age groups: 18–30 years, 31–40 years, 41–50 years, 51–60 years, 61–70 years, and 71 years of age or above. For the inferential analysis, the participants’ actual age was divided by 10 in order to estimate odds change per 10 years increase in age. Formal education level was dichotomized into 12 years’ education or less (reference category, representing high school or less education) versus more than 12 years’ education (representing some level of higher education). Employment status was similarly dichotomized into not working (reference category) versus working. The former category included persons being retired, unemployed, doing full-time housework, or receiving disability benefits, while the latter category included persons being employed with paid work or undergoing education.

### Statistical analyses

Data were analyzed using SPSS for Windows version 24 [40]. Initial descriptive analyses employed frequencies, percentages, means and standard deviations as appropriate. Univariate logistic regression analyses were performed, using self-diagnosed current depression as outcome and each of the independent variables entered separately: age, gender, education level, work status, GSE mean score, LOT-R mean score, extraversion score and neuroticism score. Finally, to adjust for covariance between the independent variables, the multivariate logistic regression analysis entered all of the independent variables together. As research has shown that personality traits sometimes interact to predict depression [25], we also tested for interactions in separate subsequent models. Effect sizes in single group comparisons were calculated as Cohen’s *d* [41], and in the logistic regression analysis as odds ratio (OR). The level of significance was set at *p* <  0.05.

### Ethics

The individuals gave informed consent to participate by completing the questionnaires and returning them anonymously to the researchers in a sealed envelope. The Regional ethics committee for medical and healthcare research in Oslo was consulted and, due to the anonymous data collected, no formal ethical approval was required.

## Results

### Responders

Altogether, 1792 persons (36.0%) opted to participate in the study. Due to missing data on the scales employed in the current study (listwise deletion), 108 responders were excluded, leaving a sample of 1684 participants for analysis.

### Sample characteristics

The sociodemographic characteristics, depression and scores on the employed scales (GSE, LOT-R, and EPQ) among the participants are shown in Table 1. The mean age of the participants was 52.7 years (*SD* = 16.5 years), with men (*M* = 55.3 years, *SD* = 15.8 years) being older than women (*M* = 50.5 years, *SD* = 16.7 years, *p* <  0.001, *d* = 0.30). Fifty-five percent of the sample had more than 12 years of education, and 67% were employed or undergoing education.

**Table 1:** Sociodemographic characteristics of participants (*n* = 1684)

Characteristics	Total (*n* = 1684)	Men (*n* = 787)	Women (*n* = 897)	*p*	*d*
Age group	n (%)	n (%)	n (%)		
18–30	203 (12.1)	70 (8.9)	133 (14.8)	< 0.001	
31–40	182 (10.8)	67 (8.5)	115 (12.8)		
41–50	345 (20.5)	145 (18.4)	200 (22.3)		
51–60	340 (20.2)	167 (21.2)	173 (19.3)		
61–70	374 (22.2)	206 (26.2)	168 (18.7)		
71 or above	240 (14.3)	132 (16.8)	108 (12.0)		
Education					
12 years or less	761 (45.2)	368 (46.8)	393 (43.8)	0.23	
More than 12 years	923 (54.8)	419 (53.2)	504 (56.2)		
Employment					
Working/in education	1127 (66.9)	503 (63.9)	624 (69.6)	0.01	
Not working/in education	557 (33.1)	284 (36.1)	273 (30.4)		
Depression					
Current self-diagnosed depression	136 (8.1)	48 (6.1)	88 (9.8)	0.005	
Lifetime self-diagnosed depression	487 (28.9)	185 (23.5)	302 (33.7)	< 0.001	
Psychological factors	M (SD)	M (SD)	M (SD)		
General self-efficacy	2.91 (0.6)	2.97 (0.6)	2.85 (0.6)	< 0.001	0.20
Optimism	2.89 (0.5)	2.88 (0.5)	2.89 (0.5)	0.69	0.02
Extraversion	3.9 (1.8)	3.6 (1.8)	4.1 (1.8)	< 0.001	0.28
Neuroticism	1.9 (1.9)	1.5 (1.7)	2.2 (1.9)	< 0.001	0.49

Statistical tests are *χ*^2^-tests for categorical variables and independent *t*-tests for continuous variables. Effect sizes are calculated as Cohen’s *d*

One hundred and 36 participants (8.1%) reported current depression, the proportions being higher for women than for men (9.8% vs. 6.1%, *p* <  0.01). Four hundred and 87 participants (28.9%) reported depression, previous or current (i.e., lifetime prevalence), these proportions also being higher for women than for men (33.7% vs. 23.5%, *p* <  0.001). Men had higher scores than women on general self-efficacy (*p* <  0.001), whereas women had a higher proportion in work or education (*p* = 0.01), and scored higher than men on extraversion and neuroticism (both *p* <  0.001). The latter difference showed a close to medium effect size. Men and women were not significantly different in their scores on optimism.

### Factors associated with depression

The results from the logistic regression analyses are shown in Table 2. In the unadjusted models, all the independent variables were significantly associated with the outcome. Having higher scores on neuroticism, or being female, were associated with higher risk of current depression. Higher age, higher education, being in work, higher general self-efficacy, more optimism and more extraversion were associated with lower risk of self-diagnosed depression (Table 2).

**Table 2:** Univariate and multivariate logistic regression analysis showing associations between the study variables and current self-diagnosed depression (*n* = 1684)

Independent variables	Univariate model				Multivariate model			
	B (SE)	OR	*p*	95% CI	B (SE)	OR	*p*	95% CI
Age increase in 10 years	−0.20 (0.05)	0.82	< 0.001	0.74–0.91	− 0.20 (0.08)	0.82	< 0.05	0.70–0.95
Gender	0.52 (0.19)	1.68	< 0.01	1.16–2.41	0.12 (0.23)	1.13	0.61	0.72–1.78
Education	−0.50 (0.18)	0.61	< 0.01	0.43–0.86	−0.01 (0.22)	0.99	0.97	0.64–1.54
Work status	−0.48 (0.18)	0.62	< 0.01	0.43–0.88	−0.56 (0.28)	0.57	< 0.05	0.33–0.98
General self-efficacy	−1.31 (0.14)	0.27	< 0.001	0.20–0.36	−0.40 (0.18)	0.67	< 0.05	0.47–0.96
Optimism	−1.68 (0.19)	0.19	< 0.001	0.13–0.27	−0.66 (0.24)	0.52	< 0.01	0.32–0.83
Extraversion	−0.25 (0.05)	0.78	< 0.001	0.71–0.86	−0.08 (0.06)	0.93	0.18	0.83–1.04
Neuroticism	0.80 (0.06)	2.23	< 0.001	1.98–2.51	0.68 (0.07)	1.97	< 0.001	1.72–2.25

Adjusted model parameters: Nagelkerke R^2^ = 0.38, Cox & Snell R^2^ = 0.16, Model χ^2^ = 285.38, *p* <  0.001, Hosmer-Lemeshow χ^2^ = 6.17, *p* = 0.63. Reference categories are lower age, male gender, low education, not working, and lower levels of general self-efficacy, optimism, extraversion, and neuroticism

In the multivariate model, controlling for the effects of all independent variables, five of the independent variables were still significantly associated with the outcome (Table 2). Most importantly, higher levels of neuroticism were strongly associated with higher risk of current self-diagnosed depression. Gender and education were no longer significantly associated with the outcome. However, lower odds of current self-diagnosed depression were associated with being in work, with higher age, and with higher levels of general self-efficacy, and optimism. Adding the interaction terms in separate subsequent models, the interaction between neuroticism and extraversion was statistically significant (*p* <  0.05), whereas the interaction between general self-efficacy and optimism was not. The pattern of associations, however, was not affected by including the interaction terms (data not shown).

To examine the sensitivity of our analysis, the logistic regression procedure was re-run restricting the outcome variable to “current self-diagnosed depression with help-seeking” versus all others. Among those with self-reported current depression (*n* = 136), six participants did not reveal information related to help seeking for mental complaints. Among the remaining 130 respondents, 78 (60.0%) had sought help. As shown in Table 3, this analysis revealed largely the same pattern of associations as shown in the main analysis.

**Table 3:** Multivariate logistic regression analysis showing associations between the study variables and current self-diagnosed depression with help seeking (*n* = 1684)

Independent variables	Multivariate model			
	B (SE)	OR	*p*	95% CI
Age increase in 10 years	−0.16 (0.10)	0.85	0.10	0.70–1.03
Gender	0.22 (0.30)	1.25	0.46	0.70–2.23
Education	0.38 (0.28)	1.47	0.17	0.85–2.54
Work status	−0.58 (0.34)	0.56	0.09	0.29–1.09
General self-efficacy	−0.51 (0.23)	0.60	< 0.05	0.39–0.93
Optimism	−0.34 (0.29)	0.71	0.24	0.40–1.26
Extraversion	−0.08 (0.07)	0.93	0.29	0.80–1.07
Neuroticism	0.74 (0.09)	2.09	< 0.001	1.74–2.51

Adjusted model parameters: Nagelkerke R^2^ = 0.35, Cox & Snell R^2^ = 0.11, Model χ^2^ = 183.24, *p* < 0.001, Hosmer-Lemeshow χ^2^ = 10.33, *p* = 0.24. Reference categories are lower age, male gender, low education, not working, and lower levels of general self-efficacy, optimism, extraversion, and neuroticism

## Discussion

In this study, the proportion of respondents with a self-diagnosed current depression was 8.1%. The prevalence was higher for women (9.8%) than for men (6.1%). This is similar to the depression prevalence in Norway established with structured clinical interviews in 2001 [42]: For men and women, the prevalence was 8.8% in urban areas and 9.3% in rural areas. For women, the prevalence was 12.0% in urban areas and 10.3% in rural areas. For men, the prevalence was 5.6% in urban areas and 7.6% in rural areas. Caution should be shown, however, when comparing estimates obtained with widely different methods. For example, a study of mothers of infants found that the frequency of cases with self-diagnosed depression was considerably higher than the frequency of cases with clinically diagnosed depression [29].

The proportion of help-seekers among those with self-diagnosed depression (60%) is similar to the proportion (65%) found in a Dutch study of help-seeking among persons with major depression [43]. Other studies have estimated that up to 50% of those with depression seek professional help [44, 45]. Considered together, it appears that the threshold for seeking help for depression is considerably higher than the threshold for reporting depression in a survey. As noted by Simon and co-workers [46], depressed persons in countries where prevalence is high tend to have lower levels of impairment associated with the depression, whereas depressed persons in countries where the prevalence is low tend to have higher levels of impairment. Thus, the discrepancy between the prevalence of depression and the number of persons seeking help for depression, as found in this study, may be owing to the participants’ experiencing lower levels of impairment associated with the depression. Help-seeking tends to increase with increasing severity of illness [47]. Alternatively, it may be owing to stigma related to help-seeking [43], to the knowledge and attitudes among the participants themselves [45], or to the (perceived) accessibility of appropriate healthcare services [47].

In agreement with previous international studies [15–17, 42], this study showed that the prevalence of self-diagnosed depression was higher among women than among men. A previous Norwegian population study suggested that the role of personality should be examined further in relationship with mental health problems [39]. We conducted a multivariate analysis in which personality factors were treated as covariates to current self-diagnosed depression. Our study showed that the association between female gender and depression became non-significant in the adjusted analysis, whereas most of the personality factors (general self-efficacy, optimism, and neuroticism) remained significantly associated with depression. Previous studies have suggested that the gender-depression relationship may be mediated by personality factors, in particular by higher levels of neuroticism among women [19, 20]. This would imply that the most important working mechanism behind the gender-depression association is neuroticism: Women tend to be more depressed compared to men because they are more prone to have higher levels of neuroticism, which in turn makes people more vulnerable to depression. Indeed, compared to the men, substantially higher levels of neuroticism were found among the women. Therefore, the reasoning that suggests a mediating role of neuroticism appears to be supported by the results.

**Fig. 2:**
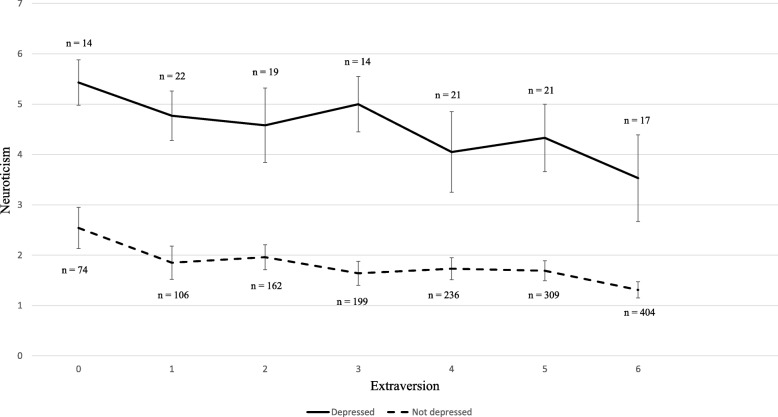
The association between neuroticism and self-diagnosed depression moderated by levels of extraversion

As suggested from the literature [9, 19], we found that neuroticism was associated with elevated odds of self-diagnosed depression. Neuroticism also interacted with extraversion, such that the association between neuroticism and self-diagnosed depression was stronger for those with lower levels of extraversion (see Fig. 2). Compared to their counterparts, those who identified as having self-diagnosed depression had higher levels of neuroticism for all levels of extraversion. However, for those with lower levels of extraversion, there was a greater difference in levels of neuroticism between those reporting to be depressed and those who did not. This appears to be in line with the findings by Vasey and co-workers [25], namely that positive emotions and liveliness (extraversion) may protect against the negative effects of negative emotionality (neuroticism). The combined impact of personality traits on depression seems to be a new line of research that deserves more attention.

On the other hand, general self-efficacy and dispositional optimism were associated with reduced risk of self-diagnosed depression. The same pattern of associations with depression was found when restricting the outcome to self-diagnosed depression with help-seeking. This solidifies our results concerning the role of these personality traits as protecting against depression. In general, however, the study supports and extends the existing literature suggesting that positive mental health outcomes are associated with higher levels of self-efficacy [48] and higher levels of optimism [7, 11].

However, in view of the cross-sectional design, we cannot rule out the possibility of “reversed causality”. Thus, one should also consider the possibility of depression affecting the individual’s perception of his or her own personality and coping resources. Those who self-identify as depressed may be more prone to score higher on neuroticism and lower on extraversion, optimism and general self-efficacy due to the negative influence of depression on self-evaluation. This question fits better with a longitudinal study, where the direction of causality can be clearly addressed.

Finally, the study suggests that higher age and being employed protects against self-diagnosed depression. In spite of frequently increasing physical health problems throughout the later stages of life, higher age may also indicate an increased capacity for reconciling with the changing opportunities and challenges in life. Such a capacity for reconciliation may serve as a buffer against self-diagnosed depression. In support of this reasoning, a study of persons with chronic obstructive pulmonary disease (mean age 65 years) found that perceiving the illness to be longer lasting, indicating reconciliation with a chronic illness progression, predicted better mental health [49]. Employment provides the person with income, social relationships, and an arena to engage in meaningful activities within a time-organized and collegial structure [50]. Unemployment may leave the person bereft of these elements, and a causal link from unemployment to depression has been shown [51]. Although the current study’s category “not employed” is not equivalent with “unemployed”, the result that employment reduces the risk of self-diagnosed depression appears to be both logical and in line with empirical research.

### Strengths and limitations

A strength of this study is the use of a large sample. In spite of a relatively low response rate, comparisons with population statistics showed that the participants’ characteristics were fairly representative of the Norwegian population [22, 30]. In addition, the combined use of several personality traits as predictors of current self-diagnosed depression makes the detected associations trustworthy.

A possible limitation is concerned with measuring depression with a single item. First, we do not know how familiar the participants were with what constitutes depression. Secondly, comparisons with other studies where depression has been assessed with clinical interviews may be difficult, and the use of single-item measures is often discouraged from a psychometric point of view. Thus, the validity of the depression measure used in this study is not fully established. However, this strict view of single-item measures has been challenged [27, 52], and single-item measures do have advantages. They are short, flexible, and easy to administer [26], and they are cost-efficient, less time consuming, and have better face validity in comparison to multi-item scales [27]. Single-item measures can be reliable, as estimated by test–retest correlations [53] and concurrence with clinical diagnosis [28]. They can correlate strongly with multi-item scales [52] and can effectively predict outcomes like mood and emotional states [54]. In our study, the main pattern of results was largely reproduced by the additional analysis where “depression with help-seeking” was used as outcome. Moreover, the prevalence estimates found in this study were similar to the previous estimates produced by using structured clinical interviews as the means of assessment [42]. This supports the validity of the main study results.

## Conclusion

The prevalence of current self-diagnosed depression was significantly higher for women than for men, and depression was associated with age, employment and psychological resources and vulnerabilities. The association between neuroticism and self-diagnosed depression was moderated by extraversion, suggesting that extraversion plays a role in depression by buffering against some of the negative effects of neuroticism. Future studies may formally investigate the validity of the single-item depression measure employed in the study. They may also combine the use of single-item self-evaluation measures of depression with established multi-item depression scales to further investigate the correspondence between these two types of measures. Future studies may also be designed as longitudinal studies so that the possible causal associations between personality, coping resources and self-diagnosed depression may be more clearly addressed.

## Abbreviations

EPQ: Eysenck personality questionnaire
GSE: General self-efficacy scale
HUNT-3: The third ‘Helseundersøkelsen i Nord-Trøndelag’ (Health Survey in Nord-Trøndelag, Norway)
LOT-R: Life orientation test-revised
OR: Odds ratio
